# Professor Koch’s Method to Cure Tuberculosis

**Published:** 1891-05

**Authors:** 


Professor Koch's Method to Cure Tuberculosis Popularly Treated by Dr. Max Birnbaum. Translated from the German by Dr. F. R. Breudicke. Milwaukee, Wis.: H. E. Haferkorn. 1891. Pp. 105. Price, paper, 75c.; cloth, $1.00.

The little work before us gives a compact *résumé* of our knowledge of tuberculosis, treated in a popular manner for physicians and the laity. Prof. Koch's first communication on the curability of tuberculosis, as published in the *Deutsche Medicinische Wochenschrift*, is appended. The frontispiece is an excellent likeness of Prof. Koch.

"THE HOME-MAKER."—*The Home-Maker* is just what the name indicates. Since Jenny June became the editor, it has steadily improved, until it is now one of the best magazines published. The April number is an excellent one, with enough illustrations to make it very interesting and readable. It abounds in bright stories, poetry, interesting articles and book notices, and all that goes to make up a magazine. Special mention should be made of the Cycle Department, devoted to the interests of federated women's clubs; a department we believe no other magazine has.

J. B. Lippincott Company will, beginning with April, issue quarterly thereafter a work entitled "International Clinics " This work will comprise the best and most practical clinical lectures on medicine, surgery, gynecology, pediatrics, dermatology, laryngology, ophthalmology, and otology, delivered in the leading medical colleges of this country, Great Britain, and Canada. These lectures have been reported by competent medical stenographers, and thoroughly revised by the professors and lecturers themselves. The object of the work is to furnish the busy practitioner and medical student with the best and most practical clinical instruction, in concise form. Each volume will consist of over 350 octavo pages, illustrated with photographic reproductions of important cases.

---

Life Among the Lepers.—Sister Rose Gertrude has written another article for *The Ladies' Home Journal* for June, on "What It is to Be a Leper," in which she gives a clear glimpse of leper life in Molakai; how the disease is contracted; how it is treated and cured, and how the lepers live in their exile.

---

Over 15,000 Manuscripts Sent to One Magazine, of which not 200 Were Accepted.—Mr. Bok, the Editor of *The Ladies' Home Journal*, recently gave some interesting figures relative to the manuscripts received by his magazine during 1890. Owing to its departments and peculiar character, the *Journal* probably receives more manuscripts than any magazine published. Mr. Bok says that he received at his office a total number of 15,205 manuscripts. Of these, 2,280 were poems; 1,746 stories and 11,179 miscellaneous articles. Of the poems, 66 were accepted; of the stories, only 21, and of the articles 410, of which latter, however, over 300 were solicited articles. Thus, it will be seen that of the entire 15,000 manuscripts only 497 were accepted; a trifle over three per cent. Deducting from this the 300 accepted articles written at the Editor's solicitation, the net percentage of unsolicited manuscripts accepted is brought down to 197, or a little more than one per cent. Statistics such as these show how much utter trash is being written, and the number of persons writing who ought to be employing their time at something else and better.

